# The efficacy and safety of Sacubitril/Valsartan on pulmonary hypertension in hemodialysis patients

**DOI:** 10.3389/fmed.2022.1055330

**Published:** 2022-11-29

**Authors:** Cong Zhao, Yanhong Guo, Yulin Wang, Liuwei Wang, Lu Yu, Yan Liang, Zihan Zhai, Lin Tang

**Affiliations:** Department of Nephrology, First Affiliated Hospital of Zhengzhou University, Zhengzhou, China

**Keywords:** Sacubitril/Valsartan, pulmonary hypertension, hemodialysis, left ventricular function, efficacy

## Abstract

**Background:**

Pulmonary hypertension (PH) is a common complication of end-stage renal disease which is associated with adverse outcomes including all-cause mortality and cardiovascular events. Recent studies have demonstrated that Sacubitril/Valsartan (Sac/Val) as an enkephalinase inhibitor and angiotensin II receptor blocker could reduce pulmonary artery systolic pressure (PASP) and improve the prognosis of patients with heart failure. However, whether Sac/Val is effective in hemodialysis (HD) patients with PH is essentially unknown. In this retrospective study, we aimed to evaluate the efficacy and safety of Sac/Val in the treatment of PH in HD patients.

**Methods:**

A total of 122 HD patients with PH were divided into Sac/Val group (*n* = 71) and ARBs group (*n* = 51) based on the treatment regimen. The PASP, other cardiac parameters measured by echocardiography, and cardiac biomarkers including N-terminal fragment of BNP (NT-proBNP) and cardiac troponin I (cTnI) were observed at baseline and 3 months after treatment.

**Results:**

There were no significant differences in the baseline characteristics between the two groups. PASP decreased significantly from 45(38, 54) to 28(21, 40) mmHg in Sac/Val group (*p* < 0.001). PASP reduced from 41(37, 51) to 34(27, 44) mmHg in ARBs group (*p* < 0.001), and the decrease was more pronounced in the Sac/Val group (*p* < 0.001). In addition, improvements in the right atrial diameter (RAD), left ventricular diameter (LVD), left ventricular posterior wall thickness (LVPWT), left atrial diameter (LAD), pulmonary artery diameter (PAD), left ventricular end-diastolic volume (LVEDV), left ventricular end-systolic volume (LVESV), left ventricular ejection fraction (LVEF), and fractional shortening (FS) were found in Sac/Val group (p_s_ < 0.05). After 3 months, LVD, LAD, LVEDV, LVESV, LVEF, SV, and PASP were significantly improved in Sac/Val group compared with ARBs group (p_s_ <0.05). Significant reduction in NT-proBNP [35,000 (15,000, 70,000) pg/ml vs. 7,042 (3,126, 29,060) pg/ml, *p* < 0.001] and cTnI [0.056(0.031, 0.085) ng/ml vs. 0.036 (0.012, 0.056) ng/ml, *p* < 0.001) were observed in Sac/Val group. No significant differences were observed in adverse events between the two groups (p_s_ > 0.05).

**Conclusion:**

Sac/Val seems to be an efficacious regimen in PH with favorable safety and has huge prospects for treating PH in HD patients.

## Introduction

Pulmonary hypertension (PH) is a pathophysiological condition characterized by abnormally elevated pulmonary arterial pressure caused by a variety of causes, and its occurrence and development are closely related to pulmonary vascular remodeling. Current evidence suggests that the incidence of PH in patients undergoing hemodialysis (HD) ranges from 19 to 69% (1). Several risk factors including volume overload, arteriovenous fistula, congestive heart failure, endothelial dysfunction, renal anemia, and metabolic disorders are implicated in PH among HD patients (2–4).

Serious PH leads to right ventricular failure and death. Right ventricular failure is the leading cause of death, accounting for 70% patients with PH with 33–38% mortality rate 3 years post diagnosis (5). PH is associated with an increased risk of all-cause mortality among HD patients. A meta-analysis of 7,112 patients showed that the risk of all-cause death in HD patients with PH was 2.3 times higher than those without PH (6, 7).

The conventional drugs for the treatment of PH mainly target pulmonary vasodilation, including endothelial receptor antagonists, phosphordiesterase inhibitors, and prostacyclin agonists (8). Pulmonary vasodilators cannot effectively control the progression of PH, although they can improve the clinical symptoms. Moreover, the use of pulmonary vasodilators is limited due to the high incidence of heart failure in HD patients. Further innovations are desperately needed in the treatment of PH.

Christof Burgdorf et al. had found that Sacubitril/Valsartan (Sac/Val) improved PH in patients with heart failure with preserved ejection fraction (HFpEF) (9). However, no studies on the role of Sac/Val in HD patients with PH were reported at present. In this retrospective study, we evaluated the effects of Sac/Val on PH in HD patients for the first time, so as to hope to provide a theoretical basis for the treatment of PH.

## Materials and methods

This is a retrospective single-center study. Seventy-one HD patients with PH using Sac/Val were enrolled from The First Affiliated Hospital of Zhengzhou University from June 2019 to December 2021. As a control group, we collected 51 HD patients with PH receiving angiotensin II type 1 receptor blockers (ARBs) at the same time. All patients enrolled met the following inclusion criteria: I. All patients were older than 18 years; II. All patients were diagnosed as PH (10); III. All patients accepted HD for more than 3 months and three times a week for 4 h according to Kidney Disease Outcomes Quality Initiative (KDOQI) (11); IV. All patients enrolled completed 3 months of follow up and had complete clinical data. Patients who met the following exclusion criteria were not included in this study: I. Patients were with cardiopulmonary diseases including obstructive sleep apnea, rheumatic heart disease, chronic obstructive pulmonary disease, lung cancer, and myocardial infarction; II. Patients were with connective tissue diseases, including systemic lupus erythematosus and Sjogren syndrome; III. Patients had problems including inadequate dialysis, hemodynamic instability, and volume overload. IV. Patients were in acute phase of infection or heart failure; IV. Patients were unable to take medication regularly; V. Patients were allergic to pharmaceutical ingredients. This study was reviewed and approved by The First Affiliated Hospital of Zhengzhou University Ethics Review Committee (2022-KY-1156). Written informed consent for participation was not required for this study in accordance with the national legislation and the institutional requirements.

All patients received basic treatments including controlling blood pressure, regulating electrolyte disturbance, and rectifying anemia. A low dose of Sac/Val and ARBs were initiated and titrated gradually to the highest tolerated dose determined by the physician in charge to maintain hemodynamic stability. The median dose of Sac/Val prescribed was 100 mg twice daily. The blood flow was kept between 200 and 300 mL/min depending on vascular access conditions of the patients.

Patient demographic characteristics such as sex, age, dialysis way, duration of dialysis, residual renal function (RRF), and self-reported history were collected. Echocardiographic parameters and cardiac biomarkers such as N-terminal fragment of BNP (NT-proBNP) and cardiac troponin I (cTnI) were evaluated at baseline and follow-up. Pulmonary artery systolic pressure (PASP) >35 mmHg measured by echocardiography is diagnosed as PH (10). Echocardiography was finished within 24 h of the completion of dialysis by two experienced sonographers from our hospital independently. Adverse events were recorded during the 3-month follow-up. The 24-h urine volume was collected to estimate the RRF of hemodialysis patient (12). Participants in two groups were divided respectively depending on presence of RRF, which was defined as a 24-h urine sample volume of ≥ 100 ml. Hyperkalemia is defined with a serum potassium level > 5.5 mmol/L. Blood pressure below 90/60 mmHg is diagnosed as hypotension.

Statistical analysis was performed with SPSS 24.0 (SPSS Inc., Chicago, IL, USA). Continuous variables were expressed as means ± standard deviation. Categorical variables were expressed as counts and percentages. Continuous variables were compared by Kruskal-Wallis test or *T*-test, while categorical variables were compared by chi-square tests or Fisher's exact tests as appropriate. The pre- and post-treatment comparisons within the same group were performed *via* paired sample *t*-test or Wilcoxon signed ranks test. All probabilities were two-tailed, and the significance level was set at 0.05.

## Results

### Baseline characteristics between the two groups

A total of 122 HD patients were identified as having PH. The mean age of the patients treated with Sac/Val was 49.13 ± 15.40 years with a male/female ratio of 45/26. The age of the patients treated with ARBs was 46.80 ± 12.25 years with a male/female ratio of 30/21. Duration of dialysis of the participants was 21 (12, 39) months in Sac/Val group and 24 (12, 36) months in ARBs group. No significant differences were identified in age, sex, duration of dialysis, dialysis access, systolic blood pressure, diastolic blood pressure, RRF, or self-reported history between the two groups. Comparison of laboratory data and echocardiographic characteristics between the two groups were summarized in Tables 1, 2. There were no differences in laboratory data including hemoglobin, hematocrit, serum potassium, serum calcium, serum phosphorus, serum albumin, and parathyroid hormone between the Sac/Val and ARBs groups (Table 1). Pulmonary artery systolic pressure (PASP) of the patients was 45 (38, 54) mmHg in the Sac/Val group and 41 (37, 51) mmHg in the ARBs group. No significant differences were noted in terms of right ventricular diameter (RVD), right atrial diameter (RAD), left ventricular diameter (LVD), left ventricular posterior wall thickness (LVPWT), left atrial diameter (LAD), pulmonary artery diameter (PAD), left ventricular end-diastolic volume (LVEDV), left ventricular end-systolic volume (LVESV), left ventricular ejection fraction (LVEF), stroke volume (SV), fractional shortening (FS), PASP, NT-proBNP, and cTnI (Table 2).

**Table 1:** Clinical characteristics of patients at baseline.

**Characteristics**	**Sac/Val** **(*n* = 71)**	**ARBs** **(*n* = 51)**	***P-value***
**Sex (** ***n*** **)**			0.610
Male	45 (63%)	30 (59%)	
Female	26 (37%)	21 (41%)	
Age (y)	49.13 ± 15.40	46.80 ± 12.25	0.408
SBP (mmHg)	150 (136, 160)	152 (133, 167)	0.712
DBP (mmHg)	86 (78, 97)	89 (80, 96)	0.745
**Dialysis access (n)**			0.541
A-V fistula	36 (51%)	23 (45%)	
Long term CVC	35 (49%)	28 (55%)	
Duration of dialysis (m)	21 (12, 39)	24 (12, 36)	0.848
**RRF (** ***n*** **)**			0.409
Yes	36 (50.7%)	22 (43.1%)	
No	35 (49.3%)	29 (56.9%)	
**Self-reported** **history (*****n*****)**			
Hypertension	69 (97%)	48 (94%)	0.704
Diabetes	17 (24%)	13 (25%)	0.845
CAD	13 (18%)	8 (16%)	0.705
Atrial fibrillation	7 (10%)	4 (8%)	0.950
Hb (g/L)	95.48 ± 22.66	91.08 ± 25.82	0.320
HCT (%)	0.29 ± 0.07	0.28 ± 0.08	0.250
Serum potassium (mmol/L)	4.47 (3.97, 5.24)	4.54 (4.18, 4.87)	0.850
Serum calcium (mmol/L)	2.16 ± 0.27	2.13 ± 0.23	0.485
Serum phosphorus (mmol/L)	1.85 (1.41, 2.35)	1.97 (1.34, 2.44)	0.518
Serum albumin (g/L)	38.50 ± 5.47	37.75 ± 6.47	0.490
PTH (pg/ml)	310 (204, 400)	275 (140, 451)	0.252

Sac/Val, Sacubitril/Valsartan; ARBs, Angiotensin receptor blockers; A-V fistula, arterio-venous fistula; SBP, systolic blood pressure; DBP, diastolic blood pressure; CVC, central venous catheter; RRF, residual renal function; CAD, coronary heart disease; Hb, hemoglobin; HCT, hematocrit; PTH, parathyroid hormone.

**Table 2:** Echocardiographic parameters and cardiac biomarkers at baseline in two groups.

**Parameters**	**Sac/Val** **(*n* = 71)**	**ARBs** **(*n* = 41)**	***P-value***
RVD (mm)	17 (16, 19)	16 (15, 18)	0.115
RAD (mm)	40 (33, 45)	37 (34, 43)	0.366
LVD (mm)	54 (50, 58)	52 (47, 58)	0.094
LVPWT (mm)	11 (10, 13)	10 (9, 11)	0.983
LAD (mm)	54 (50, 58)	39 (36, 44)	0.113
PAD (mm)	21 (20, 22)	20 (20, 22)	0.243
LVEDV (ml)	144 (118, 183)	129 (111, 149)	0.079
LVESV (ml)	63 (45, 85)	51 (42, 71)	0.053
LVEF (%)	60 (50, 64)	61 (55, 64)	0.225
SV (ml)	83 (68, 97)	74 (65, 92)	0.098
FS (%)	31 (26, 35)	33 (29,35)	0.155
PASP (mmHg)	45 (38, 54)	41 (37, 51)	0.105
NT-proBNP (pg/ml)	35,000 (15,000, 70,000)	33,754 (15,000, 42,832)	0.413
cTnI (ng/ml)	0.056 (0.031, 0.085)	0.056 (0.014, 0.085)	0.514

RVD, right ventricular diameter; RAD, right atrial diameter; LVD, left ventricular diameter; LVPWT, left ventricular posterior wall thickness; LAD, left atrial diameter; PAD, pulmonary artery diameter; LVEDV, left ventricular end-diastolic volume; LVESV, left ventricular end-systolic volume; LVEF, left ventricular ejection fraction; SV, stroke volume; FS, fractional shortening; PASP, pulmonary artery systolic pressure; NT-proBNP, N-terminal fragment of BNP; cTnI, cardiac troponin I.

### The pre- and post-treatment comparisons within the same group

From baseline to the end of follow-up, PASP was significantly reduced in Sac/Val group, starting from 45 (38, 54) mmHg reduced to 28 (21, 40) mmHg at 3 months follow-up (*p* < 0.001). PASP was reduced from 41 (37, 51) mmHg at baseline to 34 (27, 44) mmHg at 3 months follow-up in ARBs group (*p* < 0.001). There were significant differences in NT-proBNP [35,000 (15,000, 70,000) vs. 7,042 (3,126, 29,060) pg/ml, *p* < 0.001] and cTnI [0.056 (0.031, 0.085) vs. 0.036 (0.012, 0.056) ng/ml, *p* < 0.001) between baseline and 3 months follow-up in the Sac/Val group, and a similar phenomenon was observed in ARBs group. At 3 months follow-up, improvements in RAD, LVD, LVPWT, LAD, PAD, LVEDV, LVESV, LVEF, and FS were observed in Sac/Val group compared to baseline (*p* = 0.038, *p* < 0.001, *p* = 0.009, *p* < 0.001, *p* = 0.029, *p* = 0.003, *p* < 0.001, *p* < 0.001, *p* = 0.001, respectively). Improvements in RAD, RVD, LVD, LVPWT, LAD, PAD, LVEDV, LVESV, LVEF, SV and FS were not found in ARBs group (Table 3).

**Table 3:** Echocardiographic parameters and cardiac biomarkers at baseline and follow-up in two groups.

**Parameters**	**Baseline**	**Follow-up**	***P-value***
**RVD (mm)**			
Sac/Val	17 (16, 19)	16 (16, 18)	0.385
ARBs	16 (15, 18)	17 (16, 18)	0.087
**RAD (mm)**			
Sac/Val	40 (33, 45)	35 (32, 43)	0.038
ARBs	37 (34, 43)	35 (32, 44)	0.904
**LVD (mm)**			
Sac/Val	54 (50, 58)	52 (47, 55)	< 0.001
ARBs	52 (47, 58)	51 (47, 57)	0.705
**LVPWT (mm)**			
Sac/Val	11 (10, 13)	11 (9, 12)	0.009
ARBs	10 (9, 11)	12 (10, 13)	0.691
**LAD (mm)**			
Sac/Val	41 (37, 46)	38 (31, 43)	< 0.001
ARBs	39 (36, 44)	39 (34, 44)	0.466
**PAD (mm)**			
Sac/Val	21 (20, 22)	20 (20, 22)	0.029
ARBs	20 (20, 22)	20 (20, 22)	0.451
**LVEDV (ml)**			
Sac/Val	144 (118, 183)	130 (102, 149)	0.003
ARBs	129 (111, 149)	131 (103, 167)	0.746
**LVESV (ml)**			
Sac/Val	63 (45, 85)	50 (37, 60)	< 0.001
ARBs	51 (42, 71)	44 (36.5, 62.5)	0.571
**LVEF (%)**			
Sac/Val	60 (50, 64)	63 (60, 64)	< 0.001
ARBs	61 (55, 64)	63 (57, 64)	0.322
**SV (ml)**			
Sac/Val	83(68,97)	77 (64, 92)	0.072
ARBs	74 (65, 92)	76 (66, 90)	0.297
**FS (%)**			
Sac/Val	31 (26, 35)	34 (32, 35)	0.001
ARBs	33 (29, 35)	34 (31, 35)	0.278
**PASP (mmHg)**			
Sac/Val	45 (38, 54)	28 (21, 40)	< 0.001
ARBs	41 (37, 51)	34 (27, 44)	< 0.001
**NT-proBNP (pg/ml)**			
Sac/Val	35,000 (15,000, 70,000)	7,042 (3,126, 29,060)	< 0.001
ARBs	33,754 (15,000, 42,832)	18,862 (7,260, 35,000)	0.047
**cTnI (ng/ml)**			
Sac/Val	0.056 (0.031, 0.085)	0.036 (0.012, 0.056)	< 0.001
ARBs	0.056 (0.014, 0.085)	0.027 (0.012, 0.047)	0.003

### Differences between the two groups after treatment

The level of PASP was significantly lower in the Sac/Val group compared to the ARBs group at the end of follow-up. It showed that there were significant differences in the change of LVD, LAD, LVEDV, LVESV, LVEF, SV, PASP and NT-proBNP between the two groups (*p* = 0.048, *p* = 0.010, *p* = 0.019, *p* = 0.032, *p* = 0.039, *p* = 0.037, *p* < 0.001, *p* = 0.023, respectively). After 3 months, the patients in Sac/Val group had a higher LVEF. Significant differences in the change of RVD, RAD, LVPWT, PAD, and FS were not observed between the Sac/Val and ARBs groups (ps>0.05). This study showed that cTnI decreased significant in both groups, but no difference in the level of cTnI was observed between the two groups (Table 4).

**Table 4:** Changes of echocardiographic parameters and cardiac biomarkers in two groups.

**Parameters**	**Sac/Val** **(*n* = 71)**	**ARBs** **(*n* = 41)**	***P-value***
RVD (mm)	0 (−3, 2)	1 (−1, 2)	0.101
RAD (mm)	−2 (−8, 5)	1 (−7, 6)	0.241
LVD (mm)	−2.72 ± 5.33	−0.22 ± 7.01	0.048
LVPWT (mm)	−0.4 (−2, 1)	0 (−1, 1)	0.172
LAD (mm)	−3.65 ± 6.85	−0.59 ± 5.71	0.010
PAD (mm)	0 (−2, 1)	0 (−2, 1)	0.305
LVEDV (ml)	−15 (−38, 9)	3 (−23, 27)	0.019
LVESV (ml)	−12.34 ± 25.01	−1.37 ± 23.49	0.032
LVEF (%)	3 (−2, 10)	0 (−3, 5)	0.039
SV (ml)	−5.41 ± 24.16	3.8 ± 23.37	0.037
FS (%)	1 (−1, 7)	1 (−2, 3)	0.141
PASP (mmHg)	−15 (−21, −9)	−8 (−16, 2)	< 0.001
NT-proBNP (pg/ml)	−16,725 (−42,370, −622)	−6,023 (−26,494, 8,866)	0.023
cTnI (ng/ml)	−0.015 (−0.051, 0)	−0.028 (−0.067, 0.003)	0.928

### Adverse events

Hyperkalemia was observed in 9 patients (12.7%) of the Sac/Val group, and in 6 patients (11.8%) of the ARBs group (*p* = 0.880). Six patients (8.5%) had hypotension and 2 patient (2.8%) had dizziness in Sac/Val group, while 4 patient (7.8%) had hypotension and 1 patient (2.0%) had dizziness in ARBs group. None of the patients enrolled referred tussiculation and angioedema symptoms. One case of hepatic dysfunction was reported in ARBs group. In total, 17 patients (24.0%) in Sac/Val group and 12 patients (23.5%) in ARBs group suffered an adverse event. There were no significant differences in the overall incidence of adverse events between the two groups (*p* = 0.661). None of the enrolled patients discontinued the treatment due to adverse effects (Table 5).

**Table 5:** Adverse events during 3 months of follow-up.

	**Sac/Val** **(*n* = 71)**	**ARBs** **(*n* = 51)**	***P-value***
Hyperkalemia (*n*)	9 (12.7%)	6 (11.8%)	0.880
Hypotension (*n*)	6 (8.5%)	4 (7.8%)	>0.999
Tussiculation (*n*)	0	0	
Angioedema (*n*)	0	0	
Dizziness (*n*)	2 (2.8%)	1 (2.0%)	>0.999
Hepatic dysfunction (*n*)	0	1 (2.0%)	0.418
Total (*n*)	17 (24.0%)	12 (23.5%)	0.661

## Discussion

Sac/Val is approved for hypertension and chronic heart failure with reduced ejection fraction. We unexpectedly found that Sac/Val reduced PASP among HD patients with PH in clinical work. Meanwhile, several studies showed a decrease of PASP in chronic heart failure patients with PH (9, 13), but no studies had demonstrated its role in HD patients. Therefore, we conducted the first study to explore the role of Sac/Val in HD patients with PH.

PASP can be measured by the right-heart catheterization accurately. However, right-heart catheterization is not suitable for screening and retesting due to its invasiveness. Echocardiography is convenient, non-invasive and relatively reliable for evaluating PH. Moreover, it provides key information to assess the heart structure and function. Eduardo Bossone et al. found that tricuspid regurgitation peak velocity had a high linear positive correlation with PASP measured at right-heart catheterization, the sensitivity of echocardiography in estimating PASP for detecting pulmonary hypertension ranges from 0.79 to 1.00 and specificity from 0.6 to 0.98 (14, 15).

Severe PH is manifested as chest tightness, dyspnea and other symptoms of right heart failure, which seriously affects the life quality and prognosis of patients. Multiple studies have confirmed that PH is associated with a significantly increased risk of death and cardiovascular events in ESRD, and ESRD patients on hemodialysis have a higher risk than patients with CKD stages 1–5 (7). The drugs currently approved for the treatment of PH are predominantly pulmonary vasodilators. However, they may reduce the compliance of left ventricular and increase volume overload. Due to the high incidence of left heart failure, pulmonary vasodilators are not indicated for most patients undergoing HD. There is still a lack of effective treatment measure for PH.

The pathogenesis of PH in HD patients has not been fully elucidated. At present, it was believed that chronic hypoxia contributes to pulmonary artery constriction and pulmonary vascular remodeling in PH (16). In addition, uremia is a chronic inflammatory disease with endothelial dysfunction. Serum levels of cytokines including IL-1β, TNF-α, and IL-6 were elevated in PH patients undergoing HD. Tung-Min Yu et al. considered that chronic inflammation might play an important role in the pathogenesis of PH in HD patients (17, 18). The arteriovenous fistula is the access method of choice for long-term hemodialysis, arteriovenous fistulas were all formed by end-to-side anastomosis between the cephalic vein and radial artery in this study. Available evidence indicates that arteriovenous fistula provides the foundation for the emergence and progression of PH by reducing systemic vascular resistance, increasing venous blood flow, and enhancing cardiac output (19, 20). Some researchers believed that secondary hyperparathyroidism is a cause of PH in HD patients, but two studies showed no relationship between pulmonary hypertension and parathyroid hormone on nephrotic patients undergoing HD (21, 22). Several clinical studies had suggested that PH is associated with volume overload. All patients enrolled in this study had received HD for more than 3 months. A dialysis regimen of 3 times a week for 4 h was chosen, and dry weight was adjusted each time. Human studies demonstrated that the levels of endothelin-1 were elevated in circulating plasma of patients with PH. Endothelin-1 is not only a vasoconstrictor, but leads to proliferation of many vascular cells, and plays a role in the pathogenesis of PH (23). The activation of renin-angiotensin-aldosterone system (RAAS) is another essential mechanism for the development of PH in patients undergoing HD. Angiotensin II enhances the contraction of vascular smooth muscle by increasing Ca^2+^ influx into the cell membrane and induces significant proliferation of pulmonary arterial smooth muscle. Angiotensin II is widely acknowledged to be associated with the progression of disease and risk of death (24, 25). In general, the formation and progression of PH involves the interplay of multiple factors and mechanisms among HD patients.

Previous studies had shown that angiotensin II type 1 receptor blockers reduced pulmonary vascular remodeling. Fried et al. had confirmed that losartan was related to ameliorate chronic, inhaled nicotine-induced PH and right ventricular remodeling (26). Lu et al. found that valsartan slowed down the development of experimental PH in mice (27).

In contrast to RAAS, the activation of natriuretic peptide system (NPS) plays a cardioprotective role in patients with PH. Natriuretic peptides expressed in the pulmonary vascular bed had been confirmed to mediate multiple pulmonary vascular effects, including vasorelaxation, antiproliferation, and reverse remodeling (28). Studies had shown that infusion of atrial natriuretic peptide (ANP) and brain natriuretic peptide (BNP) can ameliorate PH (29). Hsu et al. suggested that BNP inhibits angiotensin II-induced proliferation and migration of pulmonary vascular smooth muscle cells (30).

Sac/Val as an enkephalinase inhibitor and angiotensin II receptor blocker affects PH in a comprehensive and targeted way. Sac/Val reduced the serum level of IL-1β, TNF-α, and IL-6 in hypoxia-induced PH rats (31). It had been demonstrated that Sac/Val inhibited the activation of endothelin associated with pulmonary vasoconstriction to decrease PASP (32). Based on the above pathogenesis and previous studies, we believed that Sac/Val is expected to become a new target for the treatment of PH in patients undergoing HD.

A significant decrease in PASP was observed in Sac/Val and ARBs groups, which was consistent with previous studies. The superior effect of Sac/Val to ARBs on reducing PASP has been demonstrated, one of the key reasons is the dual inhibitory effect of Sac/Val.

In Sac/Val group, RAD, LVD, LVPWT, LAD, PAD, LVEDV, LVESV, LVEF, and FS were all significantly improved from baseline to the end of follow-up. However, it did not exhibit significant changes in RVD and SV in Sac/Val group. LVD, LAD, RAD, LVPWT, and RVD reflect cardiac structure, LVEDV, LVESV, LVEF, SV, and FS were measured to assess the left ventricular function. Significant decreases of RAD and PAD were associated with a reduction in PASP possibly. PAD can reflect the morphological changes of the heart and provide a more accurate assessment on cardiac functional damage to a certain extent (33). The above findings suggested that Sac/Val might be related to anti-ventricular remodeling and anti-heart failure. There were no significant differences in LVD, LAD, LVPWT, RAD, PAD, LVEDV, LVESV, LVEF, and FS from baseline to follow-up in ARBs group, which was inconsistent with previous studies. One possible reason was that the observation window was too short, and more exploration was warranted in the future.

BNP is the substrate of enkephalinase, and Sac/Val as an enkephalinase inhibitor may increase BNP concentrations. In terms of patients receiving Sac/Val, the level of BNP cannot reflect the severity of heart failure accurately. Therefore, BNP is not an appropriate biomarker in this study. NT proBNP derived from the cleavage of BNP precursor provides the same information as BNP with better stability. The predictive value of NT-proBNP and cTnI for the prognosis of patients with PH has been demonstrated in many studies, and a decrease in NT-proBNP indicates amelioration in PH (34–36). The research revealed that a stronger effect on lowering the level of NT-proBNP was observed in the Sac/Val group compared with the ARBs group. Significant reductions in cTnI were found in both groups. Indeed, Sac/Val both reduced PASP and improved the cardiac function.

There was no drug withdrawal due to serious adverse reactions in this study. Hypotension was observed in 6 patients (8.5%) and dizziness occurred in 2 patients (2.8%) in Sac/Val group. Hypotension and dizziness were alleviated after adjusting the dose of Sac/Val. Hyperkalemia occurred in 9 (12.7%) and 6 (11.8%) of Sac/Val and ARBs group, respectively. One case in the ARBs group suffered from hepatic dysfunction. Hyperkalemia and hepatic dysfunction quickly returned to normal level spontaneously after symptomatic treatment, and there were no significant differences in the overall incidence of adverse events between the two groups. Therefore, Sac/Val might be an effective treatment with acceptable safety in HD patients with PH. However, the high incidence of hyperkalemia and hypotension was still noteworthy, and it needed to be explored in a larger sample study in the HD population.

However, a time-dependent improvement in pulmonary vascular remodeling needs to be considered. A more significant decrease in PASP may be observed with the extension of follow-up time, and it warrants further verification in a clinical trial. Dual treatment with Sac/Val and bosentan was reported to be superior to Sac/Val alone in terms of vascular remodeling and severity in experimental PH (37). Nonetheless, no studies have been conducted on the combination of Sac/Val and other drugs for the treatment of PH in humans. Accordingly, its feasibility needs to be further confirmed.

## Conclusion

Sac/Val is superior to ARBs in PH among HD patients and is well-tolerated. To sum up, Sac/Val is expected to be a new drug for treating PH in patients undergoing HD.

## Data availability statement

The original contributions presented in the study are included in the article/supplementary material, further inquiries can be directed to the corresponding author.

## Ethics statement

The studies involving human participants were reviewed and approved by the First Affiliated Hospital of Zhengzhou University Ethics Review Committee. Written informed consent for participation was not required for this study in accordance with the national legislation and the institutional requirements.

## Author contributions

LT conceived of the idea and provided guidance and made critical revisions to the manuscript. CZ wrote the manuscript and completed the tables and figures. YG and YW contributed to organizing the database. LW, LY, YL, and ZZ carefully reviewed the manuscript. All authors contributed to the article and approved the submitted version.

## Funding

The present study was supported by the National Natural Science Foundation of China (Grant No. U1904134) and Henan Province Young and Middle-aged Health Science and Technology Innovative Talents (Leader) Project (Grant No. YXKC2020014).

## Conflict of interest

The authors declare that the research was conducted in the absence of any commercial or financial relationships that could be construed as a potential conflict of interest.

## Publisher's note

All claims expressed in this article are solely those of the authors and do not necessarily represent those of their affiliated organizations, or those of the publisher, the editors and the reviewers. Any product that may be evaluated in this article, or claim that may be made by its manufacturer, is not guaranteed or endorsed by the publisher.

## References

[1] Bolignano D Rastelli S Agarwal R Fliser D Massy Z Ortiz A. Pulmonary hypertension in CKD. Am J Kidney Dis. (2013) 61:612–22. 10.1053/j.ajkd.2012.07.029 23164943

[2] Devasahayam J Oliver T Joseph V Nambiar S Gunasekaran K. Pulmonary hypertension in end-stage renal disease. Respir Med. (2020) 164:105905. 10.1016/j.rmed.2020.105905 32094103

[3] Yigla M Abassi Z Reisner S Nakhoul F. Pulmonary hypertension in hemodialysis patients: an unrecognized threat. Semin Dial. (2006) 19:353–7. 10.1111/j.1525-139X.2006.00186.x 16970730

[4] Edmonston DL Parikh KS Rajagopal S Shaw LK Abraham D Grabner A. Pulmonary hypertension subtypes and mortality in CKD. Am J Kidney Dis. (2020) 75:713–24. 10.1053/j.ajkd.2019.08.027 31732231 PMC7183902

[5] Sharifi Kia D Benza E Bachman TN Tushak C Kim K Simon MA. Angiotensin receptor-neprilysin inhibition attenuates right ventricular remodeling in pulmonary hypertension. J Am Heart Assoc. (2020) 9:e015708. 10.1161/JAHA.119.015708 32552157 PMC7670537

[6] Travers A Farber HW Sarnak MJ. Pulmonary hypertension in chronic kidney disease. Cardiol Clin. (2021) 39:427–34. 10.1016/j.ccl.2021.04.004 34247755

[7] Tang M Batty JA Lin C Fan X Chan KE Kalim S. pulmonary hypertension, mortality, and cardiovascular disease in CKD and ESRD patients: a systematic review and meta-analysis. Am J Kidney Dis. (2018) 72:75–83. 10.1053/j.ajkd.2017.11.018 29429751

[8] McLaughlin VV Archer SL Badesch DB Barst RJ Farber HW Lindner JR. ACCF/AHA 2009 expert consensus document on pulmonary hypertension a report of the American college of cardiology foundation task force on expert consensus documents and the American heart association developed in collaboration with the American college of chest physicians; American thoracic society. Inc; and the pulmonary hypertension association. J Am Coll Cardiol. (2009) 53:1573–619. 10.1016/j.jacc.2009.01.004 19389575

[9] Burgdorf C Brockmoller J Strampe H Januszewski M Remppis BA. Reduction of pulmonary hypertension after transition to Sacubitril/Valsartan in patients with heart failure with preserved ejection fraction. Front Cardiovasc Med. (2021) 8:734697. 10.3389/fcvm.2021.734697 34692786 PMC8529008

[10] Rudski LG Lai WW Afilalo J Hua L Handschumacher MD Chandrasekaran K. Guidelines for the echocardiographic assessment of the right heart in adults: a report from the American society of echocardiography endorsed by the European association of echocardiography, a registered branch of the European society of cardiology, and the canadian society of echocardiography. J Am Soc Echocardiogr. (2010) 23:685–713. 10.1016/j.echo.2010.05.010 20620859

[11] National Kidney F. KDOQI clinical practice guideline for hemodialysis adequacy: 2015 update. Am J Kidney Dis. (2015) 66:884–930. 10.1053/j.ajkd.2015.07.015 26498416

[12] Shafi T Jaar BG Plantinga LC Fink NE Sadler JH Parekh RS. Association of residual urine output with mortality, quality of life, and inflammation in incident hemodialysis patients: the choices for healthy outcomes in caring for end-stage renal disease (choice) study. Am J Kidney Dis. (2010) 56:348–58. 10.1053/j.ajkd.2010.03.020 20605303 PMC2910835

[13] Tran JS Havakuk O McLeod JM Hwang J Kwong HY Shavelle D. Acute pulmonary pressure change after transition to Sacubitril/Valsartan in patients with heart failure reduced ejection fraction. ESC Heart Fail. (2021) 8:1706–10. 10.1002/ehf2.13225 33522140 PMC8006690

[14] Bossone E D'Andrea A D'Alto M Citro R Argiento P Ferrara F. Echocardiography in pulmonary arterial hypertension: from diagnosis to prognosis. J Am Soc Echocardiogr. (2013) 26:1–14. 10.1016/j.echo.2012.10.009 23140849

[15] Bossone E. Echocardiography in pulmonary arterial hypertension: an essential tool. Chest. (2007) 131:339–41. 10.1378/chest.06-2475 17296630

[16] Aaronson PI. Pulmonary hypertension associated with chronic hypoxia: just asic-ness? J Physiol. (2021) 599:4731–2. 10.1113/JP282325 34533831

[17] Pullamsetti S Savai R Janssen W Dahal B Seeger W Grimminger F. Inflammation, immunological reaction and role of infection in pulmonary hypertension. Clin Microbiol Infect. (2011) 17:7–14. 10.1111/j.1469-0691.2010.03285.x 20545963

[18] Yu TM Chen YH Hsu JY Sun CS Chuang YW Chen CH. Systemic inflammation is associated with pulmonary hypertension in patients undergoing haemodialysis. Nephrol Dial Transplant. (2009) 24:1946–51. 10.1093/ndt/gfn751 19164324

[19] Abassi Z Nakhoul F Khankin E Reisner S Yigla M. Pulmonary hypertension in chronic dialysis patients with arteriovenous fistula: pathogenesis and therapeutic prospective. Curr Opin Nephrol Hypertens. (2006) 15:353–60. 10.1097/01.mnh.0000232874.27846.37 16775448

[20] Beigi A Sadeghi A Khosravi A Karami M Masoudpour H. Effects of the arteriovenous fistula on pulmonary artery pressure and cardiac output in patients with chronic renal failure. J Vasc Access. (2009) 10:160–6. 10.1177/112972980901000305 19670168

[21] Amin M Fawzy A Hamid M Elhendy A. Pulmonary hypertension in patients with chronic renal failure: role of parathyroid hormone and pulmonary artery calcifications. Chest. (2003) 124:2093–7. 10.1378/chest.124.6.2093 14665485

[22] Yigla M Keidar Z Safadi I Tov N Reisner S Nakhoul F. Pulmonary calcification in hemodialysis patients: correlation with pulmonary artery pressure values. Kidney Int. (2004) 66:806–10. 10.1111/j.1523-1755.2004.00807.x 15253737

[23] Shao D Park JE Wort SJ. The role of endothelin-1 in the pathogenesis of pulmonary arterial hypertension. Pharmacol Res. (2011) 63:504–11. 10.1016/j.phrs.2011.03.003 21419223

[24] Maron BA Leopold JA. The role of the renin-angiotensin-aldosterone system in the pathobiology of pulmonary arterial hypertension (2013 grover conference series). Pulm Circ. (2014) 4:200–10. 10.1086/675984 25006439 PMC4070776

[25] de Man FS Tu L Handoko ML Rain S Ruiter G Francois C. Dysregulated renin-angiotensin-aldosterone system contributes to pulmonary arterial hypertension. Am J Respir Crit Care Med. (2012) 186:780–9. 10.1164/rccm.201203-0411OC 22859525 PMC5104838

[26] Fried ND Morris TM Whitehead A Lazartigues E Yue X Gardner JD. Angiotensin II type 1 receptor mediates pulmonary hypertension and right ventricular remodeling induced by inhaled nicotine. Am J Physiol Heart Circ Physiol. (2021) 320:H1526–H34. 10.1152/ajpheart.00883.2020 33577434 PMC8260386

[27] Lu Y Guo H Sun Y Pan X Dong J Gao D. Valsartan attenuates pulmonary hypertension via suppression of mitogen activated protein kinase signaling and matrix metalloproteinase expression in rodents. Mol Med Rep. (2017) 16:1360–8. 10.3892/mmr.2017.6706 28586065

[28] Arjona A Hsu C Wrenn D Hill N. Effects of natriuretic peptides on vascular smooth-muscle cells derived from different vascular beds. Gen Pharmacol. (1997) 28:387–92. 10.1016/S0306-3623(96)00275-3 9068978

[29] Cargill R Lipworth B. Atrial natriuretic peptide and brain natriuretic peptide in cor pulmonale. Hemodynamic and endocrine effects. Chest. (1996) 110:1220–5. 10.1378/chest.110.5.1220 8915224

[30] Hsu JH Liou SF Yang SN Wu BN Dai ZK Chen IJ. B-Type natriuretic peptide inhibits angiotensin II-induced proliferation and migration of pulmonary arterial smooth muscle cells. Pediatr Pulmonol. (2014) 49:734–44. 10.1002/ppul.22904 24167111

[31] Liu S Wang Y Lu S Hu J Zeng X Liu W. Sacubitril/Valsartan treatment relieved the progression of established pulmonary hypertension in rat model and its mechanism. Life Sci. (2021) 266:118877. 10.1016/j.lfs.2020.118877 33310048

[32] Werner F Kojonazarov B Gassner B Abesser M Schuh K Volker K. Endothelial actions of atrial natriuretic peptide prevent pulmonary hypertension in mice. Basic Res Cardiol. (2016) 111:22. 10.1007/s00395-016-0541-x 26909880 PMC4766231

[33] Edwards P Bull R Coulden R. Ct measurement of main pulmonary artery diameter. Br J Radiol. (1998) 71:1018–20. 10.1259/bjr.71.850.10211060 10211060

[34] Warwick G Thomas PS Yates DH. Biomarkers in pulmonary hypertension. Eur Respir J. (2008) 32:503–12. 10.1183/09031936.00160307 18669790

[35] Velez-Martinez M Ayers C Mishkin JD Bartolome SB Garcia CK Torres F. Association of cardiac troponin I with disease severity and outcomes in patients with pulmonary hypertension. Am J Cardiol. (2013) 111:1812–7. 10.1016/j.amjcard.2013.02.036 23540547

[36] Heresi GA Tang WH Aytekin M Hammel J Hazen SL Dweik RA. Sensitive cardiac troponin I predicts poor outcomes in pulmonary arterial hypertension. Eur Respir J. (2012) 39:939–44. 10.1183/09031936.00067011 21885398

[37] Chaumais MC Djessas MRA Thuillet R Cumont A Tu L Hebert G. Additive protective effects of Sacubitril/Valsartan and bosentan on vascular remodelling in experimental pulmonary hypertension. Cardiovasc Res. (2021) 117:1391–401. 10.1093/cvr/cvaa200 32653925

